# Children’s Development and Parental Input: Evidence From the UK Millennium Cohort Study

**DOI:** 10.1007/s13524-017-0554-6

**Published:** 2017-02-23

**Authors:** Mónica Hernández-Alava, Gurleen Popli

**Affiliations:** 1 School of Health and Related Research, University of Sheffield, Regent Court, 30 Regent Street, Sheffield, S1 4DA UK; 2 Department of Economics, University of Sheffield, 9 Mappin Street, Sheffield, S1 4DT UK

**Keywords:** Cognitive and noncognitive development, Parental investment, Home learning environment, Dynamic factor model

## Abstract

In this study, we use the UK Millennium Cohort Study to estimate a dynamic factor model of child development. Our model follows the children from birth until 7 years of age and allows for both cognitive and noncognitive abilities in children. We find a significant self-productivity effect in both cognitive and noncognitive development, as well as some evidence of dynamic dependence across different abilities. The activities that parents carry out with children at home (parental investment) have a significant effect on children’s development; we find substantial evidence of two distinct latent parental investment variables with differential effects across the two abilities.

**Electronic supplementary material:**

The online version of this article (doi:10.1007/s13524-017-0554-6) contains supplementary material, which is available to authorized users.

## Introduction

The critical importance of the first few years of a child’s life is widely recognized. Research has shown that the levels of cognitive and noncognitive development in the early years influence schooling decisions, employment and wages, teenage pregnancy, smoking, participation in illegal activities, and incarceration (Blanden et al. 2007; Heckman et al. 2006), and also drive in part health inequalities in adulthood (Conti et al. 2010). The highest priority recommendation of the Marmot Review (Marmot 2010:20) is to “give every child the best start in life.”^1^ The document setting out the UK government policy framework (Department for Education and Department of Health 2011) emphasized the fundamental importance of children’s development during the foundation years so that children are ready for school and can take full advantage and fulfill their potential. At the same time, the review stressed the influence of parenting behavior and the impact of the home learning environment on children’s development in those early years.

Differences between children’s abilities start to form very early in life. By the time children reach school age, wide differences in their abilities emerge, and evidence has shown that children from disadvantaged households tend to fare worse in terms of cognitive and behavioral development (Heckman 2006; Hobcraft and Kiernan 2010; Kiernan and Huerta 2008; Shonkoff and Phillips 2000). These early gaps are highly persistent over time, with disadvantaged children having lower life-coping abilities (Carneiro et al. 2005; Cunha et al. 2006; Feinstein 2000; Neal and Johnson 1996).

Furthermore, research has shown that child development as early as the age of 7 predicts many important adult outcomes (Almond and Currie 2011). Using data from the children of the National Longitudinal Survey of Youth (NLSY Children and Young Adult), McLeod and Kaiser (2004) showed that behavioral and emotional problems at ages 6–8 significantly diminish the probability of receiving a high school diploma and college enrollment. Similarly, using the data from the 1958 (British) National Child Development Study, Currie and Thomas (1999) showed that test scores measured as early as age 7 have significant effects on future educational and labor market outcomes; for example, children in the lowest quartile of reading test scores at age 7 have 20 % lower wages at age 33. Skill development is a dynamic process that builds on earlier skill levels. Different skills are intertwined, thus fostering higher levels of complementary skills in the future (Knudsen et al. 2006). Heckman et al. (2006) showed that both cognitive and noncognitive skills affect schooling decisions and earnings; noncognitive skills raise wages directly through effects on productivity as well as indirectly via schooling decisions (with higher noncognitive skills leading to higher schooling).

The skill developmental process not only depends on genetics but also builds on experiences beginning as early as the prenatal period (Currie 2011; Currie and Moretti 2007). One important element of those experiences is the parental input. There is a long history of relevant work in various fields, for example, in developmental psychology (Bergeman and Plomin 1988; Bradley and Corwyn 2006; Bradley et al. 1989), epidemiology (Byford et al. 2012), sociology (Kiernan and Huerta 2008; Mercy and Steelman 1982), child health and development (Lugo-Gil and Tamis-LeMonda 2008; Melhuish et al. 2008), and more recently in economics (Aizer and Cunha 2012; Cunha and Heckman 2008; Currie 2009; Ermisch 2008). The emphasis of this research, and important common finding across disciplines, has been on the importance of the parenting quality and the home environment defined in terms of the quality of stimulation and support available to the child. Parental investment in children has been found to have its greatest impact on cognitive development in the early years but at a later stage for noncognitive development (Cunha and Heckman 2008; Cunha et al. 2010).

In the UK, a number of studies (Ermisch 2008; Kelly et al. 2011; Kiernan and Mensah 2009, 2011; Schoon et al. 2010, 2012) have shown that parental input plays a significant role in explaining child development. Ermisch (2008) used the Millennium Cohort Study data to show that much of the difference in child development (at age 3) by parents’ socioeconomic status (SES) can be explained by parents’ actions in terms of educational activities and parenting style. Kelly et al. (2011), using the same data (for ages 3 and 5), also showed that much of the gap in the child development by household income can be explained by the home learning environment, family routines, and psychosocial environment, more so for noncognitive development than for cognitive development. Kiernan and Mensah (2011) showed that children (age 5) from disadvantaged (defined as low income) families who experienced positive parenting^2^ were more likely to show a good level of achievement in school. However, although the mediating effect was large, accounting for the quality of parenting still left a large unexplained gap in the effects of disadvantage.

It is in this early period that interventions to mitigate disadvantage and enhance child development are likely to be most effective (Heckman 2006; Knudsen et al. 2006; Shonkoff and Phillips 2000). However, designing effective policy interventions requires an understanding of the complex dynamic interactions between the development of children’s skills, both cognitive and behavioral, and their home environment.

In this article, we estimate a model of cognitive and noncognitive (behavioral) development of children from birth up to the age of 7. We use longitudinal data from the UK Millennium Cohort Study (MCS), which has been following a cohort of children born in 2000–2001. Using the framework of Cunha and Heckman (2008), we estimate a dynamic factor model of skill formation, allowing for the cognitive and noncognitive skills to be interrelated and evolve jointly over time. In our analysis, we focus on parental investment in children: we investigate in detail the different measures of parental inputs and how these relate to cognitive and noncognitive dimensions of child development. To our knowledge, our study is the first comprehensive study that models both cognitive and noncognitive ability formation in very young children in the UK, using a recent cohort of children. In addition, our article departs from the current literature in one important respect as we investigate parental input and its measures in more detail.

## A Dynamic Model of Child Development

The framework of analysis uses the model of development presented in Cunha and Heckman (2008), which is a generalization of the model by Todd and Wolpin (2007).^3^ The model formalizes the growing body of evidence that cognitive and noncognitive skills are interrelated and evolve jointly over time. The levels of cognitive and noncognitive skills depend on the initial endowments with which a child is born, the socioeconomic circumstances in which the child grows up, and the continuous influence of the parents through their investment. *Parental investment* is broadly defined as the set of activities carried out by the parents with the child and the direct contribution that these activities have on the development of the child.

More formally, let ${\boldsymbol{\uptheta}}_t={\left({\uptheta}_t^C,{\uptheta}_t^N\right)}^{\prime }$ represent the vector of latent developmental variables in period *t*; ${\uptheta}_t^C$ and ${\uptheta}_t^N$ are the stocks of latent cognitive and noncognitive skills in period *t*, respectively. Childhood can be divided in *T* periods, *t* = 0, 1 , . . . *T* – 1, not necessarily equivalent to a year. This developmental state evolves over time according to the following dynamic process:

1 $$ {\boldsymbol{\uptheta}}_{t+1}={\mathbf{A}}_t{\boldsymbol{\uptheta}}_t+{\mathbf{B}}_t{\boldsymbol{\uplambda}}_t+{\mathbf{C}}_t{\mathbf{X}}_t+{\boldsymbol{\upeta}}_t,\kern2.5em  t=1,2,\ldots, T-1, $$

where **λ**_*t*_ is a (*r* × 1) latent vector representing the parental investment at time *t* toward the development of the child. The model allows for the possibility that more than one latent factor (*r* ≠ 1) underlies parental investment. **A**_*t*_, **B**_*t*_, and **C**_*t*_ contain time-varying coefficients; **X**_*t*_ is a matrix of observed variables representing the child’s current socioeconomic environment; and **η**_*t*_ are the shocks (or innovations) to skill formation assumed to be independent across individuals and independent over time for the same individual with contemporaneous covariance matrix ${\Sigma}_{\upeta \upeta}^t$. The relationship in Eq. (1) allows for a cumulative effect of parental investment, with past investments built into the current developmental state and new investments influencing development into the next period. In addition, the latent vector of parental investment at time *t* is also a function of a matrix of observable variables, ${\mathbf{X}}_t^I$,

2 $$ {\boldsymbol{\uplambda}}_t={\mathbf{C}}_t^I{\mathbf{X}}_t^I+{\upvarsigma}_t^I,\kern2.5em  t=1,2,\ldots, T-2, $$

where ${\mathbf{C}}_t^I$ is a matrix of coefficients, and ${\upvarsigma}_t^I$ are random errors independent across individuals and over time.

We assume the following equations for the initial period of observation:

3 $$ {\boldsymbol{\uptheta}}_0={\mathbf{C}}_0{\mathbf{X}}_0+\upxi $$

4 $$ {\boldsymbol{\uplambda}}_0={\mathbf{C}}_0^I{\mathbf{X}}_0^I+{\upvarsigma}_0^I, $$

where the matrices **X**_0_ and ${\mathbf{X}}_0^I$ include variables representing family background as well as natal and prenatal circumstances and immediate postnatal factors, such as breast-feeding. These two matrices do not necessarily contain the same set of variables. ξ is the random error, with covariance matrix Σ_ξξ_.

### Model for the Measurements

Both the vector of the developmental state, **θ**_*t*_, and the vector of parental investment in the child, **λ**_*t*_, are latent variables and cannot be observed directly. However, data used for empirical analysis often contain a number of observable indicators or measures that have informational content about the latent variables of interest. In the literature, these indicators are often combined to construct indices.^4^ This is not an entirely satisfactory solution because the combinations are often ad hoc and the indicators are often measured with bias, leading to problems of measurement error (Cunha and Heckman 2008). The aim of our approach is to reduce the array of observable measures to low dimensional constructs using factor models. In this way, the model takes into account measurement error and does not impose an ad hoc combination of the measures; rather, it allows the parameters to be freely estimated.

Thus, a number of observable indicators or measures denoted by ${Y}_{i, t}^k, k\in \left\{ C, N, I\right\}, i=1,\ldots,{m}_t^k$ are used as imperfect measures of the unobserved latent variables of interest, with ${m}_t^k$ $\left({m}_t^k\ge 2\right)$ denoting the number of indicators of each latent variable at time *t*. These measures can be continuous or categorical. Assuming that the outcome measures for cognitive and noncognitive development are continuous and the measures used to identify parental investment are categorical, the measurement system can be written as follows:

5 $$ {Y}_t^k={\mathbf{D}}_t^k{\uptheta}_t^k+{\mathbf{G}}_t^k{\mathbf{Z}}_t^k+{\upvarepsilon}_t^k\kern2.5em  k\in \left\{ C, N\right\} $$

6 $$ {\left({Y}_t^I\right)}^{\ast }={\mathbf{D}}_t^I{\boldsymbol{\uplambda}}_t+{\mathbf{G}}_t^I{\mathbf{Z}}_t^I+{\upvarepsilon}_t^I $$

7 $$ {y}_{j, t}^I= r\ \mathrm{if}\ {\upalpha}_{r-1}^j\ \le\ {\left({y}_{j, t}^I\right)}^{\ast}\le {\upalpha}_r^j,\kern2.5em  j=1,\ldots,{m}_t^I\ \mathrm{and}\  r=1,\ldots,{R}_t^j, $$

where ${\upalpha}_0^j=-\infty$; ${\upalpha}_{R_t^j}^j=+\infty$; ${R}_t^j$ is the number of response categories for the measure at time *t*;^5^ ${Z}_t^k, k\in \left\{ C, N, I\right\}$ are matrices of variables specific to particular measures; ${D}_t^k$ and ${G}_t^k$ are matrices of coefficients; and ${\upvarepsilon}_t^k$ are normally distributed random errors of the measurement equations (measurement errors) with a diagonal covariance matrix ${\Sigma}_{\upvarepsilon \upvarepsilon}^t$. Thus, the correlation between the observable measures at time *t* is entirely due to the underlying effect of the latent variables and the covariates ${Z}_t^k$. The matrices of coefficients ${D}_t^k$ are known as the matrices of factor *t* loadings, and the relative size of these coefficients give an indication of the importance of the measure in the underlying latent variable. Note that Eqs. (5) and (6) are equivalent and differ only in the link function used. However, the latent variables identified play different roles in the developmental process given by Eq. (1). The latent variable in Eq. (6)—the parental investment λ_*t*_—is exclusively an input into the production function, whereas the latent variables in Eq. (5)—the developmental states ${\uptheta}_t^k$—are both outputs in this period and inputs in the next period.

The assumption that parental investment is uncorrelated with η_*t*_ can be seen as a strong assumption. Parents observe external shocks, **η**_*t*_, that affect the child’s current ability for which the researchers neither observe nor have information (Todd and Wolpin 2007). Further, parental responses to these external shocks can either magnify or mitigate the impact these shocks have on child’s development; Almond and Currie (2011:1326) refer to these as “responsive investments.” To allow for responsive parental investments, we relax the assumption that **λ**_*t*_ is independent of **η**_*t*_. We consider a parental investment equation given as follows:

8 $$ {\boldsymbol{\uplambda}}_t={\mathbf{C}}_t^I{\mathbf{X}}_t^I+{\mathbf{C}}_t^{\upeta}{\upeta}_t+{\upupsilon}_t^I\kern2.5em  t=1,2,\ldots, T-2, $$

where ${\upupsilon}_t^I$ are random errors that are independent across individuals and over time and independent of η_*t*_. ${\mathbf{C}}_t^{\upeta}$ is a matrix of coefficients that captures parental response to the shocks; the direction of this response can be either reinforcing or compensatory.^6^

## Identification, Estimation, and Diagnostic Statistics

We identify the parameters in the system given by Eqs. (1)–(7) using the following assumptions:

- *Assumption 1:* The error terms **η**_*t*_, ${\upvarsigma}_t^I$, ξ, and ${\upvarsigma}_0^I$ are independent of **θ**_*t*_, **λ**_*t*_, **X**_*t*_, ${\mathbf{X}}_t^I$, **X**_0_, and ${\mathbf{X}}_0^I$.
- *Assumption 2:* The error terms **η**_*t*_, ${\upvarsigma}_t^I$, ξ, and ${\upvarsigma}_0^I$ are independent.
- *Assumption 3:* ${\upvarepsilon}_{j, t}^k$ is mean zero and independent of (θ_*t*_, λ_*t*_) for *t* ∈ {0, . . . , *T*}; $j\in \left\{0,\dots, {m}_t^k\right\}$; and *k* ∈ {*C*, *N*, *I*}.
- *Assumption 4:* ${\upvarepsilon}_{j, t}^k$ and *k* ∈ {*C*, *N*, *I*} is independent across agents and over time for *t* ∈ {0, . . . , *T*}; $j\in \left\{0,\dots, {m}_t^k\right\}$; and *k* ∈ {*C*, *N*, *I*}.
- *Assumption 5:* ${\upvarepsilon}_{j, t}^k$ is independent of ${\upvarepsilon}_{j, t}^I$ for *t* ∈ {0, . . . , *T*}; $j\in \left\{0,\dots, {m}_t^k\right\}$; *l* , *k* ∈ {*C*, *N*, *I*}; such that *l* ≠ *k*.

Assumptions 1 and 2 state that after we condition on both the observable variables (*X*_*t*_, ${X}_t^I$, *X*_0_, and ${X}_0^I$) and the unobservable variables (the level of children’s cognitive and noncognitive development (θ_*t*_) and parental investment (λ_*t*_)), there is no endogeneity and no remaining common unobservable characteristics across the equations. Assumptions 3–5 are the classical measurement error assumptions. They state that the correlations among the measurement variables can be attributed to the common effect of the observable variables in the matrices ${Z}_t^k, k\in \left\{ C, N, I\right\}$, and the unobservable characteristics given by the levels of children’s cognitive and noncognitive development, θ_*t*_, and parental investment, λ_*t*_. Any remaining unexplained components of the measurement variables are independent. These are the same assumptions as in Cunha and Heckman (2008:747), who proved identification of the model. Further, all random errors are assumed to be normally distributed.

To estimate the model with responsive parental investment (Eq. (8)), we relax the assumption that λ_*t*_ is independent of η_*t*_ (from Assumption 1). Further, we need to assume that a set of variables exists that affects parental investment and not child development; other assumptions still hold. We acknowledge here that it is difficult to identify factors/variables that have an effect only on parental investment and not child development (i.e., satisfy the necessary exclusion restriction).

We use exploratory factor analysis (EFA) to find the smallest number of interpretable factors that are needed to explain the correlations among the measurement variables. To determine the number of factors, we rely on several descriptive values and diagnostics. First, the root mean squared error of approximation (RMSEA) shows the amount of unexplained variance. RMSEA ranges from 0 to 1, with smaller values indicating better fit; acceptable model fit requires RMSEA to be significantly less than 0.05. Second, we use the comparative fit index (CFI) and the Tucker-Lewis reliability index (TLI), they both capture the discrepancy between the hypothesized model and the model that does not allow for any correlation between the model variables, adjusting for the sample size and the number of parameters estimated. Both CFI and TLI range from 0 to 1, with larger values indicating better model fit. Third, we use standardized root mean squared residual (SRMR), which is the standardized difference between the observed correlation and the predicted correlation; it allows for no penalty for the model complexity, and a value less than 0.08 is usually considered a good fit. In addition, the number of eigenvalues above 1 are sometimes used as an indication of the number of factors in the analysis. See Kline (2015) for a full discussion of the diagnostic statistics.

The dynamic factor model given by Eqs. (1)–(7) is estimated using the mean and variance adjusted weighted least squares (WLSMV) estimator. WLSMV has been shown to be a robust and a preferred estimator in the presence of dichotomous, ordinal, or continuous indicators for latent variables as long as the sample size is above 1,000 (Flora and Curran 2004; Muthén 1983, 1984); in our application, many of these indicators are either dichotomous or ordinal variables (see next section). All estimation is done in Mplus v. 7.3 (Muthén and Muthén 2010).

## Data

The Millennium Cohort Study (MCS) is a longitudinal cohort survey that follows the families of approximately 19,000 children born in 2000–2001 in the UK. Its initial design oversampled families living in areas with high proportions of ethnic minorities in England, areas where child poverty was high, and the three smaller countries of the UK (Wales, Scotland, and Northern Ireland). For a more thorough discussion of the MCS sampling design, see Plewis (2007). Given the stratified design of the survey, we use sampling weights in all the analyses reported in this article.

The data are collected via both direct interview and by self-completion. In the majority of cases, the main individual providing information about the household and child is the mother (or mother figure), but fathers/father figures are also interviewed.^7^ Five waves are available for 2001–2002, 2003–2004, 2006, 2008, and 2012 when the children were 9 months, 3 years, 5 years, 7 years, and 12 years old, respectively. In our analysis, we use the first four waves. A guide to the data sets can be found in Hansen (2012).

Originally, 20,646 families were contacted, of which 18,552 families responded (almost a 90 % response rate). The total number of cohort members (children) in the first wave was 18,818, including 246 sets of twins and 10 sets of triplets. In Wave 2, some new families were added to the survey; however, these new families cannot be included in our analysis because the information at 9 months is missing. Attrition reduces the number of families available for longitudinal analysis to 11,721 and 11,889 children, including 142 and 8 sets of twins and triplets, respectively.^8^ We excluded from the analysis all twins and triplets, babies admitted to a special care unit immediately after they were born, and babies with birth weight under 2,500 g. The final usable sample contains 9,602 children.

### Cognitive Development

A wide range of measures of cognitive ability are used in the MCS data set, although not all of them are used in each wave of the survey. The following measures described correspond to ${Y}_T^C$ in Eq. (5).

Cognitive development at age 9 months is measured using the Denver Developmental Screening Tests (DDST), which is an assessment widely used for examining the development of children from birth to age 6 years (Frankenburg and Dodds 1967; Frankenburg et al. 1992). The MCS uses a subset of the 125 items in DDST that covers three areas: fine motor function, gross motor functions, and communicative gestures. In the DDST, a baby is classified as having a delay in an item if s/he is unable to perform a task that 90 % of babies in the same age group can. If a baby shows delays in more than one item in an area, s/he is classified as having a delay in that area. This classification is based on answers from the main respondent of the survey.

Early development delays in motor functions have been shown to be highly predictive of later cognitive functions.^9^ However, it might be argued that the items in the DDST capture other concepts in addition to cognitive development. In the MCS, these are the only items that potentially have some informational content to allow identification of the cognitive development at this early age. Measurement error is likely to be large for these measures, but our modeling strategy—which strips out measurement error—will help as long as the items in the test have enough informational content on cognitive development. If the identified latent variable includes more than just cognitive development, the coefficient of cognitive development in the first wave of the developmental equation will be biased. However, the coefficients in subsequent periods will be unaffected because each period of latent cognitive development is identified using period-specific measures.

A range of standard tests of cognitive development in the other waves was administered to the child by a trained interviewer: British Ability Scales Naming Vocabulary (BAS-NV); BAS Word Reading (BAS-WR); BAS Picture Similarity (BAS-PS); BAS Pattern Construction (BAS-PC); Bracken School Readiness Assessment (BSRA); and Progress in Maths (PiM) test.

BAS-NV is a verbal scale that assesses spoken vocabulary. This test was administered to the children at ages 3 and 5 years. In BAS-PC, the child constructs a design by putting together flat squares or solid cubes with black and yellow patterns on each side. The child’s score is based on both speed and accuracy in the task. This test was administered at ages 5 and 7 years. The BAS-PS test assesses pictorial reasoning; the test was administered only at age 3 years. Finally, in the BAS-WR, the child reads aloud a series of words presented on a card. This test is an age-appropriate version of BAS-NV and was administered to the children when they were 7 years old. Further details on BAS tests can be found in Elliott et al. (1996, 1997).

BSRA is used to assess the conceptual development of young children across a wide range of categories in separate subtests. The MCS employs six of the subtests that specifically evaluate colors, letters, numbers/counting, sizes, comparisons, and shapes. The BSRA test result used is a composite score based on the total number of correct answers across all six subtests. This test was administered when the children were 3 years old. For information on the BSRA, see Bracken (2002).

Children’s numerical and analytical skills at age 7 years were assessed using a variant of the National Foundation for Educational Research (NFER) standard Progress in Maths (PiM) test in which children are examined on a range of tasks covering numbers, shape, space, measures, and data.

Table S1 in Online Resource 1 shows sample summary statistics of these cognitive measures. The measures at the age of 9 months are binary and show very low proportions of children with motor function delays. Delays in communicative gestures appear more common in the sample. The scores of the cognitive tests at all other ages except the PiM test have been standardized and are age-adjusted in three-month intervals. The sample means of these tests are all above 50, showing a slightly higher score than the norming groups used in the BAS tests to normalize the test scores. The data have more missing values when the tests were administered to children at 3 years old than at other ages.^10^

### Noncognitive Development

Noncognitive or behavioral development at 9 months is measured using selected items from the Carey Infant Temperament scale (Carey 1972; Carey and McDevitt 1978), which capture the temperament of the children (reported by the mother) across three dimensions: mood, adaptability to new situations, and regularity. Each dimension includes a range of questions, and a total score for each of the three dimensions is obtained by adding the individual scores. High scores on the first two dimensions indicate distress and withdrawal; high scores on the last dimension indicate regularity. A child is classified as being “difficult” if his/her score on a dimension is lower than the average score for the cohort.

The noncognitive abilities of the children in the rest of the waves are assessed using the Strength and Difficulties Questionnaire (SDQ) (Goodman 1997). In MCS, the SDQ is filled out by the mother. It comprises 25 questions designed to capture the behavioral attributes of 2- to 17-year-olds.^11^ The 25 questions are then grouped to assess children on five scales:

1. *Emotional Symptoms Scale:* complains of headaches/stomach aches/sickness, often seems worried, often unhappy, nervous, or clingy in new situations, easily scared.
2. *Conduct Problems Scale:* often has temper tantrums, is generally obedient, fights with or bullies other children, can be spiteful to others, is often argumentative with adults.^12^
3. *Hyperactivity Scale:* restless, overactive, cannot stay still for long, constantly fidgeting, easily distracted, can stop and think before acting, sees tasks through to the end. (Responses to the last two items are reverse coded.)
4. *Peer Problems Scale:* tends to play alone, has at least one good friend, generally liked by other children, picked on or bullied by other children, gets on better with adults.
5. *Pro-social Scale:* considerate of others’ feelings, shares readily with others, helpful if someone is hurt, upset, or ill, kind to younger children, often volunteers to help others.

A higher number indicates worse behavioral problems for the first four scales, and the reverse is true for the pro-social scale. The first four scales are often combined to obtain a total difficulties score for the child, and it has been argued that the pro-social scale captures a different concept: “[T]he absence of prosocial behaviours is conceptually different from the presence of psychological difficulties“ (Goodman 1997:582). For this reason, the pro-social scale is excluded from the present analysis.

The bottom panel of Table S1 in Online Resource 1 presents summary statistics of the measures of noncognitive development in our sample. The hyperactivity, emotional symptoms, and peer problems scales are relatively stable over time with perhaps a small tendency for the mean to increase at the age of 7. In contrast, there is a clear movement toward fewer conduct problems as children get older with a significant drop between the ages of 3 and 5 (mean from 3.317 to 2.477), but it tends to stabilize between the ages of 5 and 7.

### Parental Investment

Parental investment at 9 months is measured using the mother’s attitudes toward childrearing. Responses to four questions about the importance for development of talking to the baby, cuddling the baby, stimulating the baby, and having a regular sleeping and eating time for the baby are used.

Responses of the mother to a wide range of questions are used at the ages of 3 and 5 to measure parental investment. The majority of the questions are identical in both waves, but the wording of some of the questions changes in order to reflect the developmental stage. For example, when the children are 3, mothers are asked about the frequency with which their child is helped with the alphabet. At age 5, they are asked instead about the frequency with which their child is helped with reading and (separately) with writing.

The questions cover a wide range of activities that parents may carry out with their children. These span activities which are directly related to preschool/school (for example, “How often does someone at home help (cohort child’s name) with numbers, counting and adding up?”) and other leisure activities indirectly related (such as, “How often do you draw, paint or make things with (cohort child’s name)?”). Many of the questions are not specific about the person doing the activity with the child, but some are. One such question is, “How often do you read to (cohort child’s name)?,” which is asked separately of both the mother and the father. This question gives us information about the degree of the father’s involvement in these parenting activities. There are also more general questions about everyday routines, such as regular bedtimes and hours spent watching television.

Table S2 in Online Resource 1 shows the sample summary statistics and the coding of the different measures. The means of the answers to these questions are similar at 3 and 5 years, suggesting that parenting styles are quite persistent over time. The only exception is the downward trend in the mean of the frequency of drawing/painting activities, which might reflect the children’s increasing independence in choosing these types of activities as they grow up.

### Covariates

A number of covariates are used in the analysis. To control for differences in the starting developmental position of children, Eq. (3), we use birth weight, the age of the mother at birth,^13^ level of education of the mother (a dummy variable for an NVQ level 4 or higher), and parental SES (NS-SEC 5 classes). Birth weight is included as a proxy for genetic endowments (Del Bono et al. 2012); mother’s age at birth, mother’s education, and parental SES is included to capture any early disadvantage that the child might face given that young mothers often come from disadvantaged backgrounds that they pass on to their children (Hawkes and Joshi 2012).

The initial level of parental investment, Eq. (4), is a function of number of months that the baby was breast-fed, mother’s educational level, and the household composition measured by the number of siblings in the family and the absence of a partner in the household. Mother’s education is included to capture the finding in the literature that educated parents, especially mothers, tend to systematically spend (invest) more time with (in) their children (Guryan et al. 2008). Evidence from the literature suggests that larger families can have a negative impact on child development because this limits the resources (financial and time) available in the family (Black et al. 2005). Information on the number of siblings in the household, along with a dummy variable for a single-parent household, is included to capture the resource constraints that the family might face. These same covariates (other than months the baby was breast-fed) are also used in the latent parental investment, Eq. (2), in each period.

Covariates, gender, and ethnicity of the child, in the measurement Eqs. (5) and (6), capture systematic differences in the observed measures for the same level of the latent variable. Meltzer et al. (2000) found that more behavioral problems are reported for boys, and Goodman (1997) suggested using a different (higher) threshold for boys. The evidence on differences in reporting according to ethnicity is mixed. In a UK study, Hackett and Hackett (1993) found that Gujarati parents have a more stringent concept than English parents of what constitutes acceptable behavior. Studies such as Miner and Clarke-Stewart (2008), Atzaba-Poria et al. (2004), and Zwirs et al. (2006) found differences in reporting according to ethnicity; however, Goodman and Goodman (2011) reported no significant differences. To allow for differences in reporting, two dummy variables—one for male, and one for white ethnic background^14^—are included in the measurement equations for noncognitive ability.

The measurement equations for cognitive ability include the age of the child when the assessment was made in all periods. Even though the cognitive measures used are defined relative to age groups, these age groups are defined in three-month intervals and on a norming sample different to the MCS sample; therefore, age should be included as a control variable (Connelly 2013). Other variables included in the cognitive measurement equations at 3, 5, and 7 years are a dummy variable for white ethnic background and a set of dummy variables identifying children who speak only English at home, English with some other language, or other language but no English.

Table S3 in Online Resource 1 presents summary statistics of all the covariates. The sample is split equally between boys and girls; the majority of the children are of a white background and speak only English at home. The average age of the mother at birth is 29, and it ranges from 13 to 48. In terms of SES, the largest group is in managerial/professional occupations, followed by the baseline group, semi-routine/routine. The number of mothers with an NVQ of 4 or higher remains unchanged in the first two waves, with a small increase in the third wave. Single-parent households are a relatively small group, and this group tends to become more numerous with time. The number of siblings, as expected, also tends to increase with time.

## Results

This section discusses the estimation results. The initial period of observation (*t* = 0 in the model in the earlier section, A Dynamic Model of Child Development) corresponds to 9 months of age and includes data and variables relating to the time when the child was born. The next three periods (*t* = 1, 2, 3) correspond to ages 3, 5, and 7.

### Measurement Equations: Child Ability

Table 1 (panel A, first column) and Table 2 (panel A, first column) report the parameter estimates of the matrices ${\mathbf{D}}_0^k\left( k\in \left\{ C, N\right\}\right)$ and the measurement Eq. (5) at 9 months for the cognitive and noncognitive latent variables, respectively. These parameters are the factor loadings and represent the influence of the latent variables on the observed measures.^15^ The signs are as expected. Higher levels of cognitive development (the latent variable on Table 1) lead to lower probabilities of gross and fine motor function and communicative gestures delays. Similarly, higher levels of noncognitive development (the latent variable on Table 2) decrease the probabilities of showing low positive mood, distress to novelty, and irregularity. Columns 2–4 of Tables 1 (panel A) and 2 (panel A) report the estimated coefficients of the measurement equations of cognitive and noncognitive development variables, respectively, at ages 3, 5, and 7. The factor loadings are significant and have the expected signs: higher levels of cognitive development are associated with better scores in the tests of cognitive development in all waves, and higher levels of noncognitive development are associated with lower scores in the SDQ scales indicating fewer behavioral problems.

**Table 1:** Parameter estimates of the measurement equations for the cognitive latent variable

Measure	Covariates (latent variables)			
	${\uptheta}_0^C$	${\uptheta}_1^C$	$\kern2.5em {\uptheta}_2^C$	${\uptheta}_3^C$
Panel A				
Gross motor function delay^a^	–1			
	––			
Fine motor function delay^a^	–0.938**			
	(0.170)			
Communicative gestures delay^a^	–0.625**			
	(0.129)			
BSR composite standard score		1.238**		
		(0.047)		
BAS Naming Vocabulary		1	1	
		––	––	
BAS Picture Similarity			0.784**	
			(0.033 )	
BAS Pattern Construction			0.994**	0.944**
			(0.042)	(0.042)
BAS Word Reading				1
				––
Numerical & Analytical Skills				1.174**
				(0.043)
	Covariates (observed variables)			
	Child’s Age	White	English and Other	No English
Panel B				
Gross motor function delay^a^	4.730**			
	(0.430)			
Fine motor function delay^a^	–0.654			
	(0.453)			
Communicative gestures delay^a^	–3.273**			
	(0.325)			
BSR composite standard score	0.823**	0.375**	–0.690**	–1.639**
	(0.128)	(0.140)	(0.151)	(0.224)
BAS Naming Vocabulary (age 3)	0.612**	0.727**	–1.488**	–2.703**
	(0.146)	(0.125)	(0.139)	(0.290)
BAS Naming Vocabulary (age 5)	–0.106	0.586**	–1.606**	–2.352**
	(0.197)	(0.119)	(0.150)	(0.251)
BAS Picture Similarity	0.252	0.028	–0.175	–0.477*
	(0.238)	(0.127)	(0.148)	(0.231)
BAS Pattern Construction (age 5)	–1.235**	0.195	–0.611**	–0.606*
	(0.233)	(0.145)	(0.161)	(0.259)
BAS Pattern Construction (age 7)	0.236	0.775**	–0.114	–0.152
	(0.187)	(0.141)	(0.154)	(0.262)
BAS Word Reading	–0.120	–0.471**	0.224	0.191
	(0.198)	(0.156)	(0.158)	(0.239)
Numerical & Analytical Skills	1.725**	–0.021	–0.404*	–0.452^†^
	(0.221)	(0.148)	(0.156)	(0.264)

*Note:* Standard errors are shown in parentheses.

^a^Dummy variable.

^†^ *p* < .10; *p < .05; **p < .01

**Table 2:** Parameter estimates of the measurement equations for the noncognitive latent variable

Measure	Covariates (latent variables)			
	${\uptheta}_0^{NC}$	${\uptheta}_1^{NC}$	${\uptheta}_2^{NC}$	${\uptheta}_3^{NC}$
Panel A				
Low positive mood^a^	–1			
	––			
Distress to novelty^a^	–1.954**			
	(0.461)			
Irregularity^a^	–1.815**			
	(0.385)			
Hyperactivity Scale^b^		–0.971**	–1.302**	–1.266**
		(0.038)	(0.046)	(0.054)
Emotion Symptoms Scale^b^		–0.937**	–1.860**	–2.122**
		(0.034)	(0.057)	(0.063)
Conduct Problems^b^		–1	–1	–1
		––	––	––
Peer Problems^b^		–0.507**	–0.912**	–0.958**
		(0.023)	(0.036)	(0.036)
	Covariates (observed variables)			
	White	Male		
Panel B				
Hyperactivity Scale^b^ (age 3)	–0.046	0.195**		
	(0.082)	(0.043)		
Emotion Symptoms Scale^b^ (age 3)	–0.190**	0.013		
	(0.058)	(0.032)		
Conduct Problems^b^ (age 3)	–0.074	0.092*		
	(0.082)	(0.038)		
Peer Problems^b^ (age 3)	–0.268**	0.016		
	(0.052)	(0.033)		
Hyperactivity Scale^b^ (age 5)	–0.209**	0.106**		
	(0.069)	(0.032)		
Emotion Symptoms Scale^b^ (age 5)	–0.229**	–0.081*		
	(0.073)	(0.037)		
Conduct Problems^b^ (age 5)	0.012	0.086**		
	(0.048)	(0.024)		
Peer Problems^b^ (age 5)	–0.133**	0.045^†^		
	(0.054)	(0.025)		
Hyperactivity Scale^b^ (age 7)	–0.144*	0.209**		
	(0.069)	(0.036)		
Emotion Symptoms Scale^b^ (age 7)	–0.073	–0.118**		
	(0.081)	(0.039)		
Conduct Problems^b^ (age 7)	0.060	0.089**		
	(0.048)	(0.024)		
Peer Problems^b^ (age 7)	–0.150**	0.073**		
	(0.046)	(0.026)		

*Note:* Standard errors are shown in parentheses.

^a^Dummy variable.

^b^Higher values indicate worse behavioral problems.

^†^ *p* < .10; *p < .05; **p < .01

Panel B of Tables 1 and 2 reports the estimates of the matrices ${G}_t^k\left( k\in \left\{ C, N\right\}\right)$, the coefficients associated with the covariates in the measurement Eq. (5). These estimated coefficients show systematic differences in the measures for the same level of the latent variable; this is referred to as measurement *noninvariance*. Panel B in Table 1 shows that for the same level of development, children who speak a second language at home tend to score lower in cognitive tests than children who speak only English at home. This could signal that children who speak a second language at home might have difficulties with the cognitive test itself even if their level of cognitive development is the same as children who speak only English. The effect, however, disappears as the children get older and is significant for only one of the measures of cognitive development by the time children are age 7.

Panel B in Table 2 shows that mothers tend to report higher levels of hyperactivity and conduct problems for boys and more emotional problems for girls (at 5 and 7 years) for the same level of latent noncognitive development. In addition, mothers of children of white ethnic background also report fewer peer problems and fewer emotional problems.

### Measurement Equations: Parental Investment

Table 3 reports the parameter estimates of the matrices ${\mathbf{D}}_0^I$, the measurement Eq. (6) at 9 months for the latent parental investment variable. The signs are again as expected. The probabilities of strongly agreeing with the importance of stimulating, talking to, and cuddling the baby, and regular sleeping and feeding habits increase for higher levels of the underlying latent variable. The largest loading is on the importance of talking to the baby, and the lowest is on the importance of regular sleeping and feeding times.

**Table 3:** Parameter estimates of the measurement equations of the parental investment latent variable at 9 months

Measure	λ_0_
Importance of Stimulating the Baby^a^	–1
	––
Importance of Talking to the Baby^a^	–2.961**
	(0.435)
Importance of Cuddling the Baby^a^	–1.028**
	(0.039)
Importance of Regular Sleeping/Feeding Times^a^	–0.419**
	(0.020)

*Note:* Standard errors are shown in parentheses.

^a^Coded as discrete values ranging from 0 = strongly agree to 4 = strongly disagree.

**p < .01

The measures available in the first wave are somewhat limited compared with the rest of the periods. When the children are 3 and 5 years old, the survey contains many more usable measures of parental investment, and we conduct an exploratory factor analysis to investigate the possibility that more than one latent variable underlies parental investment; results of this exercise are reported in Table 4. In both waves, we find that all the statistics and indicators point toward two latent factors in parental investment. In both cases, there are two eigenvalues above 1, the RMSEA is significantly under 0.05, both CFI and TLI are above the usual rule of thumb of 0.95, and the SRMR is also at an acceptable value.

**Table 4:** Summary results of the exploratory factor analysis for parental investment

	RMSEA	*p* < .05	CFI	TLI	SRMR
Children’s Age 3 Years					
Eigenvalues	2.106		1.484	0.924	
One factor	0.084	.000	0.646	0.504	0.090
Two factors	0.024	1.000	0.981	0.960	0.019
Three factors	0.003	1.000	1.000	1.000	0.005
Children’s Age 5 Years					
Eigenvalues	2.617		1.266	0.981	
One factor	0.067	.000	0.886	0.847	0.064
Two factors	0.034	1.000	0.980	0.961	0.027
Three factors	0.019	1.000	0.996	0.987	0.014

*Notes:* RMSEA = root mean square error of approximation. CFI = comparative fit index. TLI = Tucker-Lewis reliability index. SRMR = standardized root mean square residual.

Table 5 presents the parameter estimates ${\mathbf{D}}_t^I$ of the measurement Eq. (6) for the parental investment latent variables at ages 3 and 5 after we split the measures according to the results of the factor analysis. No additional covariates are included in the parental investment measurement equations. The first factor is a more general factor that includes activities that parents and children carry out together as well as TV watching and bedtime routines. The second factor is more focused on helping children with preschool and school-related matters.

**Table 5:** Estimated parameters of the parental investment measurement equations

Measure	Age of Child, 3 Years		Age of Child, 5 Years	
	${\uplambda}_1^{(1)}$	${\uplambda}_1^{(2)}$	${\uplambda}_2^{(1)}$	${\uplambda}_2^{(2)}$
Frequency Mother Reads to the Child	1		1	
	––		––	
Frequency Father Reads to the Child	0.535**		0.612^†^	
	(0.060)		(0.082)	
Frequency Child Taken to the Library	0.375**		0.699**	
	(0.043)		(0.103)	
Frequency Regular Bedtime	0.371**		0.801**	
	(0.042)		(0.113)	
Frequency Watching TV	–0.125**		–0.331**	
	(0.025)		(0.062)	
Frequency Child Paints/Draws at Home		1		1
		––		––
Frequency Child Helped With Alphabet		2.403**		
		(0.205)		
Frequency Child Helped With Reading				2.027**
				(0.124)
Frequency Child Helped With Writing				3.985**
				(0.253)
Frequency Child Helped With Counting/Math		4.108**		2.950**
		(0.579)		(0.160)

*Note:* Standard errors are shown in parentheses.

^†^ *p* < .10; **p < .01

Of note is that at the age of 3 years, the two largest coefficients (factor loadings) in the more general factor are in the equations for the measures of the frequency with which the mother and the father read to the child. By the age of 5, the frequency with which the mother reads to the child still has the largest factor loading, but the coefficient in the measure of the frequency with which the father reads to the child is now much smaller in relative terms. When looking at the second parental latent variable, we find that the largest factor loading at age 3 is in the measure of help with counting being overtaken by help with writing by the age of 5, following the patterns of learning over time.

Our findings of two distinct latent parental investment variables are supported by the findings in the child development literature. Yeung et al. (2002) discussed two distinct parental inputs. Consistent with our first factor, they call the first input the *family process*, which focuses on the parenting activities. Consistent with our second factor, they refer to the second input the *investment process*, whereby parents focus on the human capital of their children and invest in materials and activities to enhance the learning environment of their children.^16^

### Initial Period Latent Equations

The estimated coefficients **C**_0_ and ${\mathbf{C}}_0^I$ of the initial period Eqs. (3) and (4) are shown in Table 6. The findings are in line with previous studies. Birth weight is positively and significantly related to the level of cognitive development in the initial period. The age of the mother at birth is also significant for both cognitive and noncognitive development and exhibits a concave relationship; the maximum size effect is reached at approximately 28 years of age in the cognitive development equation and at approximately age 37 in the noncognitive development equation. The household SES at the time of the birth also shows a significant relationship to the level of development of the child with higher coefficients found for higher-SES groups.

**Table 6:** Estimated parameters of the initial latent equations

	Cognitive Development, ${\uptheta}_0^C$	Noncognitive Development, ${\uptheta}_0^{NC}$	Parental Investment, λ_0_
Birth Weight	0.136**	0.039	
	(0.021)	(0.027)	
Breast-feeding (months)			0.230**
			(0.064)
Breast-feeding (months), Squared			–0.133*
			0.064)
Age of Mother at Birth / 10	0.870**	1.202**	
	(0.197)	(0.220)	
Age of Mother at Birth / 10, Squared	–0.742**	–0.968**	
	(0.194)	(0.219)	
Parental Socioeconomic Status			
Managerial/professional	0.902**	0.651**	
	(0.072)	(0.104)	
Intermediate	0.491**	0.689**	
	(0.087	(0.095)	
Small employer/self-employed	0.455**	0.190	
	(0.106)	(0.116)	
Lower supervisors/technical	0.138	0.225*	
	(0.089)	(0.099)	
Mother NVQ of 4 or Higher	0.294**	–0.145	–0.010
	(0.097)	(0.157)	(0.088)
Number of Siblings			–0.016
			(0.034)
Single-Parent Household			0.084
			(0.059)

*Notes:* All reported coefficients are standardized. For the continuous covariates, the coefficient represents the change in the dependent variable associated with a 1 standard deviation change in the covariate. For the binary covariates, the coefficient represents the change associated with a shift in the variable from 0 to 1. Standard errors are shown in parentheses.

*p < .05; **p < .01

For the parental investment equation, the only significant coefficients are for breast-feeding, which exhibits a concave relationship.^17^ Mother’s education, number of siblings, and single parenthood are not significantly associated with parental investment when the children are very young (9 months).

### Latent Parental Investment Equations

Table 7 depicts the estimated coefficients ${\mathbf{C}}_t^I$ of the parental investment Eq. (2) at ages 3 and 5. At age 3, mothers with NVQ level 4 and higher tend to have a higher average parental investment in the child; the presence of other siblings and absence of a partner in the household has no impact on the parental investment at this age. However, by age 5 (the time when children have started the first compulsory year at school), mother’s education, the number of other siblings, and absence of a partner in the household become significant, especially for the factors related directly to school activities.

**Table 7:** Estimated parameters of the parental investment equations

	Age of Child, 3 Years		Age of Child, 5 Years	
	${\uplambda}_1^{(1)}$	${\uplambda}_1^{(2)}$	${\uplambda}_2^{(1)}$	${\uplambda}_2^{(2)}$
Mother NVQ of 4 or Higher	0.342**	0.014	0.364**	0.152*
	(0.091)	(0.089)	(0.107)	(0.073)
Number of Siblings	–0.060	–0.036	–0.055	–0.074**
	(0.046)	(0.044)	(0.043)	(0.027)
Single-Parent Household	–0.077	0.001	–0.341**	–0.119*
	(0.084)	(0.068)	(0.082)	(0.054)

*Notes:* All reported coefficients are standardized. For the continuous covariates, the coefficient represents the change in the dependent variable associated with a 1 standard deviation change in the covariate. For the binary covariates, the coefficient represents the change associated with a shift in the variable from 0 to 1. Standard errors are shown in parentheses.

*p < .05; **p < .01

### Dynamic Latent Development Variable Equations

Table 8 presents the parameter estimates **A**_*t*_, **B**_*t*_, and **C**_*t*_ of the dynamic Eq. (1). There is a significant autoregressive effect in both cognitive and noncognitive development; higher levels of cognitive (noncognitive) development foster higher levels of development in future periods. There is also some evidence of cross-equation dependence. Cognitive development increases noncognitive development in the next period, but this effect is statistically significant only when the child is age 3. Similarly, level of noncognitive development has a significant effect on future levels of cognitive development only at age 3.

**Table 8:** Parameter estimates of the developmental dynamic equations

Covariates	Age of Child, 3 Years		Age of Child, 5 Years		Age of Child, 7 Years	
	${\uptheta}_1^C$	${\uptheta}_1^N$	${\uptheta}_2^C$	${\uptheta}_2^N$	${\uptheta}_3^C$	${\uptheta}_3^N$
${\uptheta}_{t-1}^C$	0.591**	0.228**	0.759**	0.020	0.856**	0.014
	(0.047)	(0.047)	(0.018)	(0.019)	(0.014)	(0.019)
${\uptheta}_{t-1}^N$	0.118*	0.438**	0.027	0.801**	0.021	0.948**
	(0.048)	(0.046)	(0.020)	(0.015)	(0.014)	(0.012)
${\uplambda}_{t-1}^{(1)}$	0.057**	0.033^†^	0.266**	0.123**	0.197**	0.121**
	(0.019)	(0.019)	(0.021)	(0.210)	(0.025)	(0.023)
${\uplambda}_{t-1}^{(2)}$			0.131**	–0.010	0.044**	0.039*
			(0.018)	(0.017)	(0.016)	(0.018)

*Notes:* All reported coefficients are standardized. For the continuous covariates, the coefficient represents the change in the dependent variable associated with a 1 standard deviation change in the covariate. For the binary covariates, the coefficient represents the change associated with a shift in the variable from 0 to 1. Standard errors are shown in parentheses.

^†^ *p* < .10; *p < .05; **p < .01

Parental investment is a significant determinant of future cognitive and noncognitive development. The first and more general parental investment factor is significant throughout; it has a much larger standardized coefficient than the second parental investment factor, relating to school matters, in all equations. Therefore, a 1 standard deviation change in the general factor leads to a much larger relative effect in both cognitive and noncognitive development. Note that although the general parental investment factor has a larger effect on cognitive development, it also has a sizable effect on noncognitive development, thus highlighting the importance of parents and children interactions for children’s noncognitive development. The second parental investment factor related directly to helping children with school-related activities is found to be significant for only cognitive development when children start school; however, by the time children are age 7, it becomes significant for both cognitive and noncognitive development. This might be capturing the effort parents make when children start school to ensure a good start.

Table S4 in Online Resource 1 shows the estimated correlation matrix of the latent child ability. These correlations amongst the latent variables are due to the dynamics as well as the common influences of the covariates. The correlations suggest that the initial levels of development are important. The correlations between the initial level of cognitive (noncognitive) development and the levels of development at other time periods are always positive, which reflects the raw correlations that we see in the data in which children with a high level of initial cognitive (noncognitive) development are more likely to show higher levels of cognitive (noncognitive) development as they grow up. These correlations with the initial levels tend to decrease slowly over time; the correlations of the initial latent cognitive (noncognitive) development with the latent cognitive (noncognitive) development at 3, 5, and 7 years are 0.62, 0.49, and 0.45 (0.49, 0.40, and 0.38), respectively. However, the correlations between latent development in subsequent periods increase substantially; the estimated correlation between cognitive development is. 78 between ages 3 and 5, and .87 between ages 5 and 7; corresponding figures for noncognitive development are .81 and .96, respectively. These correlations are an indication of the complex nature of development and show that the starting developmental position has an important influence on the developmental path; but other continuing influences, such as the parenting activities, become very important influences as time goes by.

Last, we estimated a model with responsive parental inputs given by Eq. (8). We assume breast-feeding, number of siblings, and single-parent household as the exogenous covariates that affect parental investment but not child development.^18^ Qualitatively, the results were similar to those presented in Tables 7 and 8. We found evidence of compensatory investment by parents mitigating the effect of negative shocks. However, as suggested earlier, identification of the model was weak, leading to some large insignificant variances in some of the cognitive development equations, which have a marked effect on the standardized coefficients. Therefore, those results are not presented here but are available on request.

## Discussion

The first few years of a child’s life are widely recognized to be crucial in shaping their future. Recent research has shown that the levels of cognitive and noncognitive development influence medium-term achievements and decisions, such as schooling, and also long-term outcomes, such as employment and wages, smoking, and participation in illegal activities. Research has also shown that gaps in cognitive and noncognitive abilities start to form very early in life and are highly persistent over time, with children from disadvantaged backgrounds having on average lower levels of cognitive and noncognitive development.

We use longitudinal data from the Millennium Cohort Study to estimate a dynamic factor model of child development in the UK using the framework of Cunha and Heckman (2008). Our estimation methodology allows us to address the issue of measurement error in measuring both the ability of the child and the parental investment made in the child. The data used cover the early years of a child’s life from birth to age 7, the period when interventions to alleviate disadvantages are likely to have the largest effect. MCS is unique in collecting rich data on both children’s outcomes and their family environment, including detailed information on parental involvement with the children. To our knowledge, ours is the first comprehensive study of development in these early years for the UK using these rich data.

In line with similar research using data from other countries or data from different developmental ages, we find significant evidence of a self-productive effect in both cognitive and noncognitive development; higher levels of cognitive (noncognitive) development today foster higher levels of cognitive (noncognitive) levels in the future. The evidence of a strong self-productivity effect reiterates the importance of any policy to be targeted as early in the life of the child as possible. In addition, we find some evidence of cross-dependence between different abilities in the preschool years.

The literature has increasingly emphasized the role of parental investments in the development of children, especially in the early years. Our study contributes to this literature. Similar to other research in the area, we find that parental investment is a significant influence in children’s developmental trajectories. Although other studies assumed that parental investment can be summarized in one latent variable, the rich data on parental involvement in children available in MCS allow us to investigate the possibility of more than one factor behind our measures of parental input and find evidence of two distinct factors. One factor is more general and covers a range of activities that parents carry out with their children, such as reading to the child, in addition to usual routines and practices. This factor has a significant effect on both cognitive and noncognitive development throughout all these early years of development; within this factor, reading to the child seems to be relatively the most important input. The second parental investment factor is related to helping children with school matters, especially numeracy and written work. This latent parental input affects cognitive development after children enter formal schooling (i.e., from age 5, the first year of compulsory education in the UK) and becomes important for noncognitive development by the time children are 7 years old.

However, our analysis has limitations. The longitudinal data used in the analysis allow us to identify the stage-specific role of parental investments, but nonrandom attrition in the sample remains an issue for our analysis just like any other analysis using longitudinal data. Although parental investment is identified, its persistence is not modeled. Because persistence is high in our sample, one would expect this to reinforce the effect of policies and other external shocks.

A number of policy implications follow from our analysis and findings. Given the importance of parental investment in the children’s developmental trajectories throughout the first few years, efforts should concentrate on designing policies to help parents improve the home learning environment in the early years. Specifically, findings from our study suggest that any policy targeted toward the early years of the children should be aimed at encouraging both the general investment in children and in helping them with school-related activities. The relative importance of the general parental investment is evident from the very beginning and is likely to have higher short-term returns. Further, helping children to succeed in school is beneficial not only for their academic achievement but also potentially for their social and emotional development.

However, these policies will not be successful if they have only a temporary effect on parental investment because other influences, such as other socioeconomic circumstances, might eventually outweigh the effect. For example, our findings suggest that educated mothers tend to have higher average parental investment in the children: this is true for the general factor at all ages and holds true for the school-related factor when the children reach school age (5 years). This finding is not unique to our study. Sparked by Robert Putman’s (2016) recent book, *Our Kids*, on social mobility in the United States, Richards et al. (2016) wrote a report to explore the antecedents of social mobility in the UK. One of the key domains stressed in Putnam (2016) and explored by Richards et al. (2016) is parental time invested in children younger than 5, which Richards et al. (2016) call the “Gruffalo time” (this includes reading and talking to the children and playing with them—i.e., nonacademic activities, similar to the first factor of parental investment in this article). Similar to the findings for the United States, Richards et al. found that although over time, parents are spending more time with their children, with an increase from an average of 23 min per day in 1975 to 80 min on average per day in 2015, the size of the socioeconomic gap over this period has increased from 20 to 30 min in 1975 to 40 min per day in 2015.

In another study, Bradbury et al. (2012) conducted a cross-country (United States, UK, Australia, and Canada) comparison of school readiness of children (aged 5) with focus on their home environments and the role of public policy. They found significant inequalities in child development according to family income and mother’s education in all four countries. However, these inequalities are most pronounced in the United States, which has the least generous provisions for families with young children among all the four countries, followed by the UK. Despite many policy initiatives in the UK aimed at reducing inequalities in the formative years (e.g., extended maternity leave and universal health care), there remains a bias in favor of policies of late intervention, even when these policies are costly and of little success (Allen 2011a, 2011b).^19^

Policies designed to increase parental investment at different stages of a child’s life complemented by policies to tackle the source of initial inequalities will have a much higher likelihood of reducing the gaps in both cognitive and noncognitive development. As initial inequalities increase (as is the case in countries like the United States and UK), the interaction between family background and public policies that determine a child’s opportunities will become increasingly important and be driven by the national context, with a need for progressive public policy that benefits more the disadvantaged families (Corak 2013).

## Electronic supplementary material

ESM 1 (PDF 125 kb)

## Footnotes

1 The Marmot Review was an independent review set up in 2008, by the then Secretary of State for Health for the UK, to propose effective evidence-based strategies to reduce health inequalities in England.

2
*Positive parenting* is defined by the authors as promotion of reading and learning; relationships and interactions with the child; aspects of family organization, care, and nutrition; and disciplinary practices.

3 Although more general nonlinear models of development have been presented (Cuhna et al. 2010), here we focus on a basic linear specification.

4 For example, the home score index designed by Bradley and Caldwell (1980, 1984) is a simple summation of the response given by the main caregiver of the child to a range of items used to capture the support (learning-specific and emotional) that a child receives at home. This index has been used extensively in the literature; see Todd and Wolpin (2007) for a full discussion.

5 The outcome measures for both cognitive and noncognitive development in the initial period are binary variables. In this case, the linear Eq. (5) is replaced by a probit specification. This is analogous to the equations for the parental investment Eqs. (6) and (7) for the case of two categories $\left({R}_t^j=2\right)$ of the binary outcome.

6 See Almond and Currie (2011) for a full discussion of conditions under which parents reinforce or compensate for the negative shocks to child development.

7 For the sample analyzed in this article, 98 % of the main respondents are the biological mothers of the children; where father information is available, the majority are the biological fathers of the children, ranging from 99 % in Wave 1 to 92 % in Wave 4.

8 Refusing to participate was the biggest reason for attrition. The refusal rates are higher for the “disadvantaged” and “ethnic minority” families, relative to the “advantaged” families, across all the four countries of the UK. For details on response rates in MCS, see Ketende (2010).

9 Piek et al. (2008, 2010) found a significant predictive relationship between fine and gross motor trajectories in early childhood (ages 4 months to 4 years) and cognitive ability and levels of anxious/ depressive symptoms at school age (measured at 6 to 12 years).

10 One advantage of using the latent variable specification for child ability (both cognitive and noncognitive) and parental investment is that as long as we have data available on two measures for the latent variable for the child, s/he can be included in the analysis. For example, at age 5 we have three measures (test scores) of child’s cognitive ability (BAS-NV, BAS-PC, and BAS-PS). As long as the data are available on two of these measures, we can include the child even if the measurement on the third test score is missing.

11 There are two versions of SDQ: one for children aged 2–4, and the second for children aged 4–17 (www.sdqinfo.org). MCS uses the version for ages 2–4 in MCS2 and the version for ages 4–17 in MCS3 and MCS4. The only difference between the two versions is the wording on two questions in the Conduct Problem scale.

12 For the ages 4–17 version of SDQ, the last two questions are changed to “steals from home, school, elsewhere” and “often lies or cheats.”

13 Irrespective of the main respondent, this is the age of the biological mother of the child.

14 The vast majority (88 %) of the children in the analysis sample are white; looking across the census ethnic-6 classifications (white, Indian, Pakistani and Bangladeshi, Black and Black British, mixed, and others), there are very few observations in the individual ethnic categories; hence, we do not use these categories separately in the analysis.

15 The factor loadings can be identified only up to a scale. For each latent variable, in each period, one factor loading is normalized to 1; this is reflected in the tables.

16 In their work, Hsin and Felfe (2014) called the first factor *unstructured activities and social activities* and the second factor *structured play activities*.

17 A quadratic term in breast-feeding captures two different issues. First, although breast-feeding is beneficial to the baby, benefits of exclusive breast-feeding start to decrease after the first four to six months; evidence suggests that exclusive breast-feeding after four to six months can potentially be detrimental to child development, especially by increasing the risk of iron deficiency anemia (Fewtrell et al. 2011). Second, the MCS data show a sharp decrease in breast-feeding rates after age 4 months; at age 4 months, only 3 % of babies are breast-fed, and this drops to 0.3 % by age 6 months (Kelly and Watt 2005). These prevalence rates should be viewed in light of the fact that at the time of the first MCS survey, the UK guideline for exclusive breast-feeding was age 4 months (Sacker et al. 2006), which was later revised to age 6 months per the WHO guidelines in 2003 (WHO and UNICEF 2003).

18 Evidence from MCS shows that the association between breast-feeding and development is reduced when other factors (biological, socioeconomic, and psychological) are controlled for (Sacker et al. 2006). At the same time, breast-feeding is strongly correlated with social class (Kelly and Watt 2005). This indicates that although breast-feeding may well be associated with positive development directly, it potentially has a much stronger association with social class of the parents, thus capturing some unobserved parenting characteristics independent of child development. Justification for the use of the other variables as exogenous comes from the argument of resource constraints, discussed in the section on covariates.

19 The Allen Review (Allen 2011a, 2011b) was an independent review of Early Intervention programs requested by the UK government. It lists the 25 most successful early intervention programs in the UK, and makes recommendations for further policy actions.
