# Supplementary material for: Children’s Development and Parental Input: Evidence From the UK Millennium Cohort Study
Source: Demography. 2017 Feb 23;54(2):485–511. doi: 10.1007/s13524-017-0554-6 (PMC5371649; doi:10.1007/s13524-017-0554-6)
Supplement: Supplementary file 1 — (PDF 125 kb) [file 13524_2017_554_MOESM1_ESM.pdf]

Online Resource 1

Children's Development and Parental Input: Evidence From the UK Millennium Cohort Study

Mónica Hernández-Alava and Gurleen Popli

Table S1: Unweighted sample descriptive statistics of cognitive and noncognitive measurement variables

	Age of child											
	9 months			3 years			5 years			7 years		
	Mean	Std dev	n	Mean	Std dev	n	Mean	Std dev	n	Mean	Std dev	n
Cognitive measures												
Gross motor function delay[(1)]	0.107	0.309	9600									
Fine motor function delay[(1)]	0.124	0.330	9567									
Communicative gestures delay[(1)]	0.388	0.487	9560									
BSR composite standard score				53.440	28.400	8755						
BAS Naming Vocabulary				53.267	28.249	9154	53.006	28.240	9512			
BAS Picture Similarity							51.686	28.430	9502			
BAS Pattern Construction							52.771	28.531	9485	52.213	28.493	9447
BAS Word Reading										51.780	28.385	9339
Numerical&Analytical Skills										51.949	28.268	9473
Non-cognitive measures												
Low positive mood[(1)]	0.489	0.500	8793									
Distress to novelty[(1)]	0.444	0.497	6339									
Irregularity[(1)]	0.433	0.496	9126									
Hyperactivity Scale[(2)]				4.086	1.702	9230	4.009	1.470	9406	4.164	1.500	9413
Emotional Symptoms Scale[(2)]				1.271	1.400	9302	1.293	1.509	9439	1.448	1.694	9415
Conduct Problems Scale[(2)]				3.317	1.616	9313	2.477	1.065	9447	2.468	1.059	9431
Peer Problems Scale[(2)]				4.350	1.328	9244	4.647	1.129	9429	4.688	1.138	9412

[(1)]Dummy variable

[(2)]Higher values indicate worse behavioural problems

Table S2: Unweighted sample descriptive statistics of parental investment measurement variables.

	Age of child								
	9 months			3 years			5 years		
Measure	Mean	Std dev	n	Mean	Std dev	n	Mean	Std dev	n
Importance of stimulating baby[(1)]	1.384	0.598	9406						
Importance of talking to baby[(1)]	1.176	0.427	9408						
importance of cuddling baby[(1)]	1.172	0.439	9408						
Importance of regular sleeping/eating for baby[(1)]	1.588	0.742	9406						
Frequency mother reads to the child[(2)]				3.319	1.006	9602	3.265	0.925	9597
Frequency father reads to the child[(2)]				2.088	1.372	8302	2.014	1.315	8750
Frequency child taken to the library[(3)]				0.494	0.747	9602	0.443	0.655	9597
Frequency child paints/draws at home[(2)]				3.173	0.919	9602	1.990	0.995	9597
Frequency child helped with alphabet[(2)]				2.133	1.387	9602			
Frequency child helped with reading[(2)]							3.378	0.927	9602
Frequency child helped with writing[(2)]							2.693	1.204	9601
Frequency child helped with counting/maths[(2)]				3.162	1.052	9602	2.814	1.150	9601
Frequency regular bedtime[(4)]				2.152	0.897	9602	2.493	0.779	9601
Frequency watching TV[(5)]				1.927	0.652	9596	1.915	0.634	9594

[(1)] Coded as (1) strongly agree, (2) agree, (3) neither agree nor disagree, (4) disagree, (5) strongly disagree

[(2)] Coded as (0) not at all, (1) once/twice/less a month, (2) once/twice a week, (3) several times a week, (4) every day

[(3)] Coded as (0) never/special occasion, (1) at least once a month, (2) once a week or more

[(4)] Coded as (0) never or almost never, (1) sometimes, (2) usually, (3) always

[(5)] Coded as (0) none, (1) up to one hour, (2) between 1 and 3 hours, (3) more than 3 hours

Table S3: Unweighted descriptive statistics of sample covariates

	Mean	Std dev
Birthweight (Kg)	3.47	0.46
Male	0.50	0.50
White	0.88	0.33
Child's age - 1st wave (months)	9.19	0.50
Child's age - 2nd wave (months)	37.54	2.34
Child's age - 3rd wave (months)	62.63	2.92
Child's age - 4th wave (months)	86.78	2.99
Mother's age at birth (years)	29.20	5.61
Parental socioeconomic status		
- managerial/professional	0.48	0.50
- intermediate	0.14	0.35
- small employer/self employed	0.07	0.25
- lower supervisors/technical	0.09	0.29
- semi-routine and routine (baseline)		
Mother NVQ 4 or higher - 1st wave	0.36	0.48
Mother NVQ 4 or higher - 2nd wave	0.36	0.48
Mother NVQ 4 or higher - 3rd wave	0.39	0.49
Single parent household - 1st wave	0.11	0.31
Single parent household - 2nd wave	0.13	0.34
Single parent household - 3rd wave	0.15	0.36
Length of breastfeeding (months)	2.90	3.43
Number of siblings - 1st wave	0.92	1.01
Number of siblings - 2nd wave	1.20	1.03
Number of siblings - 3rd wave	1.39	1.02
English and other language spoken at home	0.09	0.28
No English spoken at home - other language	0.03	0.16

Table S4: Correlation matrix of latent child ability

	θ_0^C	θ_0^N	θ_1^C	θ_1^N	θ_2^C	θ_2^N	θ_3^C	θ_3^N
θ_0^C	1							
θ_0^N	0.20	1						
θ_1^C	0.62	0.24	1					
θ_1^N	0.32	0.49	0.25	1				
θ_2^C	0.49	0.20	0.78	0.22	1			
θ_2^N	0.27	0.40	0.22	0.81	0.23	1		
θ_3^C	0.45	0.19	0.68	0.21	0.87	0.22	1	
θ_3^N	0.28	0.38	0.23	0.77	0.24	0.96	0.26	1

All correlations are significant at 1% level
